# Correlation of the Rates of Solvolysis of Neopentyl Chloroformate—A Recommended Protecting Agent

**DOI:** 10.3390/ijms12021161

**Published:** 2011-02-15

**Authors:** Malcolm J. D’Souza, Shannon E. Carter, Dennis N. Kevill

**Affiliations:** 1 Department of Chemistry, Wesley College, 120 N. State Street, Dover, DE 19901, USA; 2 Department of Chemistry and Biochemistry, Northern Illinois University, DeKalb, IL 60115, USA

**Keywords:** solvolysis, 1,2-methyl shift, LFER, addition-elimination, Grunwald-Winstein equations, ionization, neopentyl chloroformate

## Abstract

The specific rates of solvolysis of neopentyl chloroformate (**1**) have been determined in 21 pure and binary solvents at 45.0 °C. In most solvents the values are essentially identical to those for ethyl and *n*-propyl chloroformates. However, in aqueous-1,1,1,3,3,3-hexafluoro-2-propanol mixtures (HFIP) rich in fluoroalcohol, **1** solvolyses appreciably faster than the other two substrates. Linear free energy relationship (LFER) comparison of the specific rates of solvolysis of **1** with those for phenyl chloroformate and those for *n*-propyl chloroformate are helpful in the mechanistic considerations, as is also the treatment in terms of the Extended Grunwald-Winstein equation. It is proposed that the faster reaction for **1** in HFIP rich solvents is due to the influence of a 1,2-methyl shift, leading to a tertiary alkyl cation, outweighing the only weak nucleophilic solvation of the cation possible in these low nucleophilicity solvents.

## 1. Introduction

Chloroformates are used in large amounts in various industrial and pharmaceutical applications, including the preparation of dyes, plastics, bulk chemicals, pharmaceuticals, and flotation agents [1,2]. In particular, they are very important reagents for the introduction of protecting groups during peptide synthesis [3]. The benzyl ester or its *p*-nitro derivative is often used for this purpose [4,5] and the bridgehead 1-adamantyl chloroformate has been found to be useful [6].

**Figure 1.**
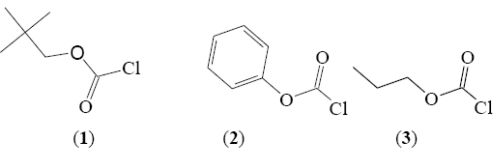
Molecular structures of neopentyl chloroformate (**1**), phenyl chloroformate (**2**), and *n*-propyl chloroformate (**3**).

For several years, we have been investigating the mechanisms available for the solvolyses of chloroformate esters; these reactions offer a model for nucleophilic substitution reactions of chloroformates in general, including their use within peptide synthesis and many other applications. The solvolyses involved in our study include those of phenyl (**2**, Figure 1) [7], *p*-methoxyphenyl [8], *p*-nitrophenyl [9], methyl [10], ethyl [11], *n*-propyl (**3**, Figure 1) [12], isopropyl [13,14], benzyl and *p*-nitrobenzyl [15,16], 2-adamantyl [17], 1-adamantyl [18], and 2,2,2-trichloro-1,1-dimethylethyl [19].

A powerful tool for investigating the mechanisms of solvolysis reactions is the Grunwald-Winstein equation [20]. The initial standard substrate was *t*-butyl chloride and the initial standard solvent was 80% ethanol-20% water (by volume at 25.0 °C) [21]. The forms of the equation parallel that of the better known Hammett equation [22] except that, instead of varying the characteristics of the substrate, we are varying the characteristics of the solvent. The one-term (original) equation was expressed as in Equation 1:

(1) $$\text{log}(k/k_{o})=\text{mY}+c$$

In the equation, *k* and *k_o_* are the specific rates of solvolysis (first-order rate coefficients) in a given solvent and in the standard solvent, respectively, *m* represents the sensitivity to changes in ionizing power (*Y*). For the standard substrate, *m* is set at unity and log(*k*/*k_o_*) then represents the *Y* scale. For any other substrate *c* is a constant (residual) term. The original *tert*-butyl chloride *Y* scale was found to include a small contribution from solvent nucleophilicity [23,24] and *Y*_X_ scales are now usually used for a leaving group X, where the scale is based on the 1-adamantyl or 2-adamantyl group being attached directly to the X being displaced in the solvolysis [25,26].

Many solvolyses are, however, bimolecular in nature, where the solvent also acts in the rate-determining step as the nucleophilic reagent. Reduced *m* values are usually observed if the one-term Equation 1 is applied to a range of compositions of a binary solvent mixture. However, considerable scatter is observed between the points obtained for the combination of several binary mixtures. It was realized early [27] that it would be necessary to incorporate a term governed by the sensitivity (*l*) to changes in solvent nucleophilicity (*N*) and Equation 2 was proposed.

(2) $$\text{log}(k/k_{o})=\text{lN}+\text{mY}+c$$

A major problem was that it was not possible to rigidly determine *N* without the sensitivity of the standard substrate solvolysis (*m* value) to changes in *Y* being known. Attempts [25] were made to arrive at a good estimate of the *m* value for the solvolyses of methyl *p*-toluenesulfonate. The solvent nucleophilicity scale usually employed (*N*_T_) is based on the solvolyses of *S*-methyldibenzothiophenium ion [28]. Here the leaving group is a large neutral dibenzothiophene molecule and ionizing power considerations can be neglected, such that the log(*k*/*k_o_*) values can be taken directly as representing an *N* scale, termed the *N*_T_ scale. Equation 2 can be written as in Equation 3:

(3) $$\text{log}(k/k_{o})={\text{lN}}_{\text{T}}+{\text{mY}}_{\text{X}}+c$$

The development of solvent nucleophilicity scales has been reviewed [20,29]. It was found that, although developed for nucleophilic attack at an sp^3^-hybridized carbon, the scale could also be applied to nucleophilic attack at the sp^2^-hybridized carbonyl carbon of acyl halides [30] and chloroformate esters [7]. The mechanism for the solvolyses of phenyl chloroformate [7,31], *p*-methoxyphenyl chloroformate [8,31] and *p*-nitrophenyl chloroformate [9] is believed to be uniformly addition-elimination across the full range of solvents usually used in Grunwald-Winstein treatments. One strong piece of evidence for the addition step being rate-determining for the reactions of haloformate esters is that the fluoroformate reacts at a similar rate to the chloroformate and, indeed, often somewhat faster [1]. This strongly indicates that the carbon-halogen bond is not broken in the rate-determining step [32].

**Scheme 1.**
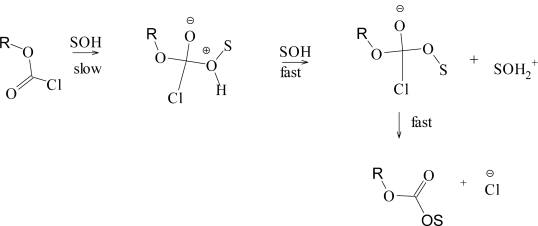
Stepwise addition-elimination mechanism through a tetrahedral intermediate for chloroformate esters.

Analyses [9,31], using Equation 3, leads to *l* values of 1.66, 1.46, and 1.58 and to *m* values of 0.56, 0.53, and 0.57, respectively. The *l*/*m* ratio can be considered as a good indicator of mechanism for attack at acyl carbon and values are obtained of 2.96, 2.75, and 2.77. Values in this range can be taken as one indicator of addition-elimination (association-dissociation), with the addition-step rate-determining (Scheme 1).

In Scheme 1, the mechanism is depicted with proton removal from the tetrahedral intermediate after the slow step. There is, however, evidence for what can be considered as a termolecular mechanism [33,34] with general base catalysis by one solvent molecule towards nucleophilic attack at an acyl carbon by a second [35–37].

**Scheme 2.**
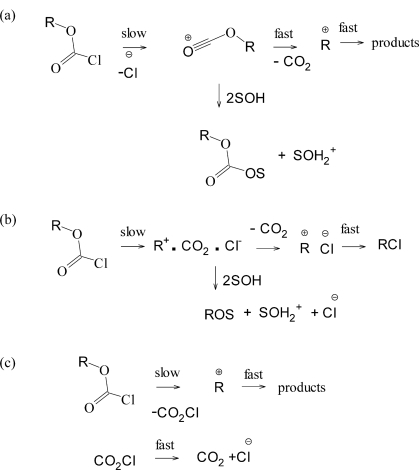
Possible unimolecular solvolytic pathways for chloroformate esters.

For methyl and primarily alkyl chloroformates, the mechanism of Scheme 1 is followed in all solvents except those of very low nucleophilicity and ionizing power, such as solvents rich in 2,2,2-trifluoroethanol (TFE) or 1,1,1,3,3,3-hexafluoro-2-propanol (HFIP). In these solvents, an ionization mechanism, assisted by nucleophilic solvation of the developing cation is believed to operate. The cation in a simple ionization will be an acyl cation but there is also evidence [18] for concerted solvolysis-decomposition such that the cation formed prior to the product formation is the alkyl cation. For the formation of relatively stable carbocations (for example, tertiary), a process is also possible to give the carbocation and the chloroformate anion, which then loses carbon dioxide. These three variants are shown in Scheme 2.

As one moves to the secondary isopropyl chloroformate, there are now appreciable ranges of solvent within which either addition-elimination or ionization is dominant [13,14] and, for the tertiary 1-adamantyl chloroformate, the major products are the decomposition product, 1-adamantyl chloride, and an ether and/or the alcohol (depending on the solvent components). All are formed by capture of the 1-adamantyl cation, formed in one of the pathways shown in Scheme 2.

Neopentyl (2,2-dimethylpropyl) chloroformate (**1**) is commercially available and its uses include that as an inert pendant protecting group in the development of model polymers, such as poly(4-neopentyloxycarbonyl)styrene, for photoresist resins [38] and as an acylating agent in the enantioselective syntheses of biologically significant compounds [39]. Its solvolysis reactions are of interest as regards their reaction mechanism. The addition-elimination reactions may be slightly retarded due to the bulk of the neopentyl group but nothing particularly unusual would be predicted. Similarly, if the ionization pathway depicted in Scheme 2 (a) is followed, no special effects should be observed.

If the solvolyses of **1** should follow either of the pathways (b) or (c) of Scheme 2 then acceleration, relative to simpler primary alkyl chloroformates, may well be observed due to the possibility of a favorable carbocation rearrangement taking place during the rate-determining process of these pathways [40,41]. These Wagner-Meerwein-type rearrangements involve a 1,2-methyl migration, leading to a more stabilized 3° alkyl carbocation as opposed to a 1° one which would have been formed in an unperturbed ionization process (Equation 4).
(4)
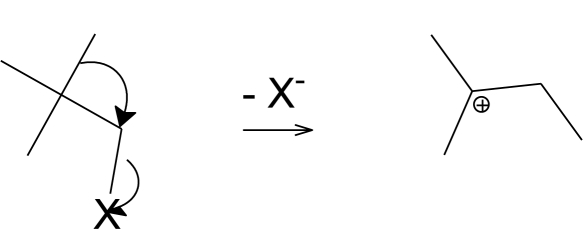


If the process of Scheme 2 (b) or (c) is followed, a faster reaction would be expected relative to the corresponding *n*-propyl reaction, where hydride migration is possible, but will only lead to the formation of a 2°carbocation (isopropyl) if it should occur.

In this study we will look at the neopentyl/*n*-Pr rate ratios for solvolyses of the chloroformate esters and at applications of the single (Equation 1) and extended (Equation 3) forms of the Grunwald-Winstein equation to the solvolytic rate coefficients (specific rates). A major aspect of this study will be to search for evidence of accelerated solvolyses of **1**, resulting from 1,2-migrations of the Wagner-Meerwein-type.

## 2. Results and Discussion

The specific rates of solvolysis of **1** at 45.0 °C are determined by monitoring amounts of acid produced in titration reactions. The results are reported in Table 1. Also presented in Table 1 are the *N*_T_ and *Y*_Cl_ values needed for the correlation analysis of the assembled data using Equation 3. The *l*, *m*, and *c* values obtained, together with the multiple correlation coefficients (*R*) and the *F*-test values are reported, together with corresponding values from the literature for solvolyses of other chloroformate esters in Table 2.

It is of interest to compare the data for **1** with that for the previously studied [12] *n*-propyl chloroformate (**3**). In this way, one can access the kinetic influence of the two β-methyl groups which are introduced from going from **3** to **1**.

A simple specific rate comparison is hampered by the present study of **1** being at 45.0 °C and the study of **3** being primarily at 25.0 °C. Fortunately, for **3**, some studies were also carried out at other temperatures, including 45.0 °C. The following specific rate ratio (*k***_1_**/*k***_3_**) are for specific rates directly determined at 45.0 °C in the indicated solvent: 100% EtOH, 1.44; 100% MeOH, 1.14; 80% EtOH, 0.93; 70% TFE, 0.89; 70% HFIP, 0.87. The rate ratios are all close to unity which would suggest that, in each solvent, the solvolysis mechanism is probably identical for the two substrates. In ethanol, methanol, and 80% ethanol, it was proposed [12] that the solvolysis of **3** were solidly in the addition-elimination camp, and such a mechanism is also reasonable for **1**. It would appear that the introduction of the two β-methyl groups into **3** has little effect as regards either the electronic or steric influence when there is a direct attack by the solvent at the acyl carbon. The observation that the specific rate ratio remains close to unity in 70% TFE and 70% HFIP, solvents where the ionization mechanism is believed to be dominant for **3**, suggest that the larger electronic effect influences to be expected within this pathway are also essentially identical. This would suggest that it is probably the pathway shown in Scheme 2 (a) which is operating, since a rate-determining formation of the alkyl cation would be expected to be favored for **1**, because of the possibility of the Wagner-Meerwein rearrangement from a 1° to a 3° cation. This argument is supported by the *k***_3_**/*k***_Et_** ratios for **3** and ethyl chloroformate in 70% and 50% HFIP also being close to unity (1.12 and 0.84, respectively). The closeness to unity of all the ratios suggest that the major stabilization is coming from nucleophilic solvation and differences in electronic influences with ethyl, *n*-propyl, or neopentyl as the alkyl group in the alkyl chloroformate can, to a close approximation, be neglected. Since **1** reacts at essentially the same rate as the other primary alkyl chloroformates, it would appear that an alkyl cation is not being formed in that rate-determining step in the solvents included in the rate comparisons. Unfortunately the fluoroalcohol-water mixtures with a larger percentage of fluoroalcohol were not studied as solvents (and reactants) at a common temperature.

**Figure 2.**
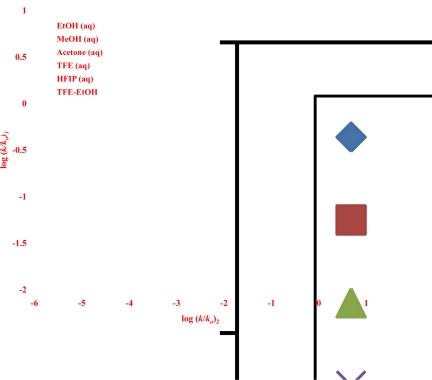
The plot of log(*k*/*k_o_*) for solvolyses of neopentyl chloroformate (**1**) at 45.0 °C against log(*k*/*k*_o_) values for solvolyses of phenyl chloroformate (**2**) at 25.0 °C.

An alternative approach is to carry out linear free energy relationship (LFER) correlations of the specific rates of solvolyses. For such an approach, while studies of the two systems at a common temperature are desirable, it is not a critical consideration. The first correlation of this type that we will consider is of the log(*k*/*k*_o_) values of **1** at 45.0 °C against the log(*k*/*k*_o_) values for phenyl chloroformate (**2**, at 25.0 °C). The *k* and *k*_o_ are defined as in Equations 1–3. Since **2** is believed to react by an addition-elimination mechanism over the full range of solvents, any contribution from additional mechanisms for the solvolysis of **1** will lead to the log(*k*/*k*_o_) values deviating upward from the plot. This type of plot has been favored as a mechanistic tool by Bentley and coworkers [42]. The plot obtained from plotting all of the data available for **1** against the corresponding data for **2** is shown in Figure 2. A good correlation is obtained in four of the six binary systems included in the plot with the aqueous fluoroalcohols deviating upwards, consistent with the observations for ethyl [11] and *n*-propyl [12] chloroformates of the superimposition onto the addition-elimination pathway of an ionization pathway, which becomes increasingly dominant as the percentage of fluoroalcohol in the solvent is increased. One can estimate from the plot that the rate for 97% HFIP is about four orders of magnitude greater than one would predict for the addition-elimination pathway. The thirteen colored-filled data points (fluoroalcohol-water mixtures excluded) represent a plot with a correlation coefficient of 0.977, slope of 0.91 ±0.06, and an intercept of −0.06 ±0.05.

**Figure 3.**
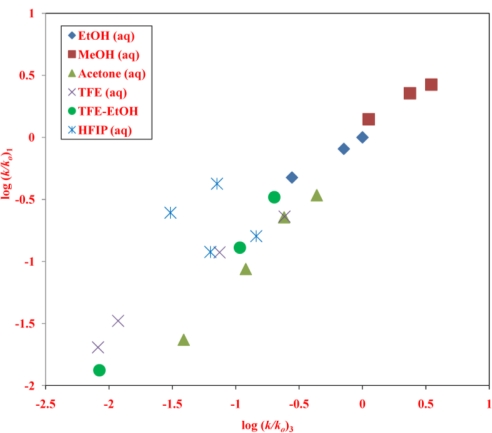
The plot of log(*k*/*k_o_*) values for solvolyses of neopentyl chloroformate (**1**) at 45.0 °C against log(*k*/*k*_o_) values for solvolyses of *n*-propyl chloroformate (**3**) at 25.0 °C.

Another worthwhile plot of this type is against the log(*k*/*k*_o_) values for *n*-propyl chloroformate (**3**). A change in mechanism from addition-elimination to ionization occurs for both **1** and **3**. If this occurs at about the same region of solvent variation, then a linear plot will still be obtained for the LFER. This plot, with the data for **1** at 45.0 °C and for **3** at 25.0 °C is shown in Figure 3 for all of the 21 solvents studied for **1**. The correlation coefficient of 0.925 is quite low and one can readily see that this is largely because the data points in 97% and 90% HFIP lie to an appreciable extent above the plot and a considerable improved correlation (*R* = 0.975), with a slope of 0.87 ± 0.05 and intercept of −0.02 ± 0.05 and with an *F*-test value of 328 is obtained by removing these two points from the correlation. The difference in behavior of **1** and **3** in this region can readily be seen from a glance at the specific rates in the HFIP-H_2_O solvents. For **3** the rates decrease from 50% to 90% HFIP and then approximately double on going to 97% HFIP, such that the rate in 97% HFIP is roughly the same as in 70% HFIP. For **1**, there is a slight decrease from 50% to 70% HFIP and then increases as one goes to 90% and onto 97% HFIP, such that the rate in 97% HFIP is about 3.5 times that in 70% HFIP.

**Figure 4.**
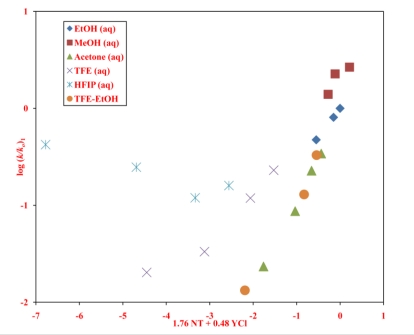
The plot of log(*k/k_o_*) values for solvolyses of neopentyl chloroformate (**1**) at 45.0 °C against 1.76 *N***_T_** + 0.48 *Y***_Cl_**. The points for HFIP-H_2_O and TFE-H_2_O mixtures are not included in the correlation. They are shown in the figure to demonstrate their appreciable deviations from the correlation line.

**Figure 5.**
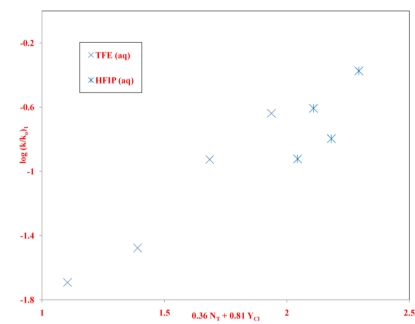
The plot of log(*k/k_o_*) values for solvolyses of neopentyl chloroformate (1) against at 45.0 °C against (0.36 *N*_T_ + 0.81 *Y*_Cl_) for HFIP-H_2_O and TFE-H_2_O mixtures.

We will postpone putting forward any rationale for this appreciable difference in behavior in the HFIP-content solvents until after a consideration of the Grunwald-Winstein correlations, using Equation 3. Previously studied primary alkyl chloroformates (ethyl and *n*-propyl) have shown the need to classify the solvents/reactants into two groups, as outlined in the introduction section. The *l* value of 1.76 ± 0.14 and *m* value of 0.48 ± 0.06, for the 13 solvents classified as going through the addition-elimination mechanism (Figure 4), give an *l*/*m* ratio of 3.67, in reasonable agreements with the corresponding values of 2.79 for **3** and 2.84 for ethyl chloroformate in this region. The other eight solvents (although a little low in number for a two-term correlation) give, in what is presumably an ionization pathway, values of 0.36 ± 0.10 for *l* and 0.48 ± 0.06 for *m* (Figure 5), for an *l*/*m* ratio of 0.44, somewhat lower than the ratio of 0.63 for **3** (only six solvents) and 0.84 for ethyl chloroformate (only seven solvents). Due to the low number of solvents care must be taken not to over interpret these values [43]. For example, if one correlates the data for **3** in the same eight solvents as for **1**, the *l*/*m* ratios rise from 0.63 to 0.73. The above values for *l* and *m* and of the *l*/*m* ratio are tabulated together with additional correlation parameters and correlation data for several other substrates in Table 2.

Since there are several possibilities for the mechanism of the ionization process, three are shown in Scheme 2, the establishment of a “standard” substrate, paralleling the choice of phenyl chloroformate (**2**) for the addition-elimination pathway, is problematic. Indeed, there is probably the need for at least two standards, for Scheme 2 pathway (a) and for (b) and/or (c). At one extreme we have the tertiary 1-adamantyl chloroformate, which except for a trace of the carbonate in 100% ethanol, gives exclusively products with loss of carbon dioxide. The observation of an *l* value of essentially zero is consistent with formation of the 1-adamantyl cation; by definition *l* is zero for solvolyses of 1-AdX substrates [23,26]. Similarly for the 2-adamantyl chloroformate, the ionization pathway is accompanied by appreciable amounts of carbonate in ethanol (88%) and lesser amount of addition-elimination in other solvents [17]. For 19 of the more ionizing and less nucleophilic solvents, and analysis in terms of Equation 3 again led to an *l* value of essentially zero, consistent with the extreme difficulty of nucleophilic participation at a 2-adamantyl carbon due to steric hindrance from hydrogens attached to the cage [26,44].

The observation of nucleophilic attack, following an addition-elimination mechanism, being the main pathway for the 2-adamantyl chloroformate in ethanol and ethanol-rich mixtures [17] shows that nucleophilic solvation would also be possible at the acyl carbon. The observation of *l* values of essentially zero for both the 1-adamantyl and 2-adamantyl etsers suggests that it is the alkyl cations, with the developing charge sheltered from solvation, which are being formed in the rate-determining step.

More typical chloroformate substrates for the ionization reaction would be the benzyl [15,16] and isopropyl esters [13,14]. These have an addition-elimination region in their solvolyses but there is also a large range of solvolyses where ionization is dominant. Values obtained from the application of Equation 3 to these solvolyses are included in Table 2. For benzyl chloroformate, the *l* value is 0.25 ±0.05 and the *m* value is 0.66 ±0.06, for an *l*/*m* ratio of 0.38 and for isopropyl chloroformate, the corresponding values are 0.28 ± 0.04, 0.59 ± 0.04, and 0.47. The corresponding *l*/*m* ratio for the ionization process of **1** is 0.44, of exactly the same magnitude.

The deviations for the solvolyses of **1** in 97% and 90% HFIP from the behavior observed for the corresponding solvolyses of **3** is believed to be due to the usually observed stabilization of the developing carbocation by nucleophilic solvation becoming sufficiently low in these very low nucleophilicity solvents that for **1** the dominant nucleophilic stabilization process becomes internal. This involves the transfer of the methyl group with its pair of bonding electrons from the β- to the α-carbon, such that the incipient carbocation has developed some tertiary character and the energy content of the transition state is reduced, leading to a faster ionization process. In principle, the *n*-propyl group can undergo a 1,2-hydride shift to give the isopropyl cation but this migration is less favored [41] and, in any event, would involve a 1°to 2° alkyl group conversion, considerably less favorable than a 1°to 3° conversion.

## 3. Experimental Section

The neopentyl chloroformate (97%, Sigma-Aldrich) was used as received. Solvents were purified and the kinetic runs carried out as described previously [7]. A substrate concentration of approximately 0.005 M in a variety of solvents was employed. For some of the runs, calculation of the specific rates of solvolysis (first-order rate coefficients) was carried out by a process in which the conventional Guggenheim treatment [45] was modified [46] so as to give an estimate of the infinity titer, which was then used to calculate for each run a series of integrated rate coefficients. The specific rates and associated standard deviations, as presented in Table 1, are obtained by averaging all of the values from, at least, duplicate runs.

Multiple regression analyses were carried out using the Excel 2007 package from the Microsoft Corporation, and the SigmaPlot 9.0 software version from Systat Software, Inc., San Jose, CA, USA, was used for the Guggenheim treatments.

## 4. Conclusions

Application of the extended Grunwald-Winstein equation (Equation 3) is further shown to be a useful probe for the investigation of the mechanism of solvolysis reactions. In some aspects the solvolyses of neopentyl chloroformate parallel those of the previously studied ethyl chloroformate and *n*-propyl chloroformate. All three substrates show regions of addition-elimination and of ionization character as the solvent is varied. For neopentyl chloroformate, this can be demonstrated either in terms of two applications of Equation 3 or by a LFER plot against phenyl chloroformate (Figure 2), a good standard substrate for the addition-elimination mechanism. The large positive deviations from the plot of the fluoroalcohol-rich solvents support the superimposition of a dominant ionization pathway in those solvents.

Particularly revealing is an identical type of LFER plot against *n*-propyl chloroformate (Figure 3). Here a good plot is obtained except for positive deviations for the data points in 97% and 90% HFIP. It is proposed that, for the other solvents, the dominant nucleophilically-driven stabilizing influence in the ionization pathway is nucleophilic solvation but this becomes sufficiently disfavored in the very weakly nucleophilic 97% and 90% HFIP, such that a Wagner-Meerwein 1,2-methyl shift in the rate-determining step, leading to the formation of the *tert*-pentyl cation, becomes the dominant cation-stabilizing influence.

## Figures and Tables

**Table 1.** Specific rates of solvolysis (*k*) of **1**, in several pure and binary solvents at 45.0 °C, and literature values for *N*_T_ and *Y*_Cl_.

**Solvent (%)***^a^*	**1 at 45.0 °C; 10^5^*k*, s^−1^*^b^***	***N*_T_***^c^*	***Y*_Cl_***^d^*
100% MeOH	47.9 ± 0.3	0.17	−1.2
90% MeOH	77.6 ± 0.6	−0.01	−0.20
80% MeOH	91.3 ± 0.6	−0.06	0.67
100% EtOH	16.3 ± 0.3	0.37	−2.50
90% EtOH	27.7 ± 0.1	0.16	−0.90
80% EtOH	34.3 ± 0.1	0.00	0.00
90% Acetone	0.804 ± 0.005	−0.35	−2.39
80% Acetone	2.99 ± 0.04	−0.37	−0.80
70% Acetone	7.81 ± 0.01	−0.42	0.17
60% Acetone	11.7 ± 0.1	−0.52	1.00
97% TFE (w/w)	0.697 ± 0.002	−3.30	2.83
90% TFE (w/w)	1.14 ± 0.01	−2.55	2.85
70% TFE (w/w)	4.07 ± 0.02	−1.98	2.96
50% TFE (w/w)	7.89 ± 0.07	−1.73	3.16
80T-20E	0.455 ± 0.002	−1.76	1.89
40T-60E	4.44 ± 0.08	−0.34	−0.48
20T-80E	11.3 ± 0.1	0.08	−1.42
97% HFIP (w/w)	14.5 ± 0.1	−5.26	5.17
90% HFIP (w/w)	8.48 ± 0.01	−3.84	4.41
70% HFIP (w/w)	4.09 ± 0.01	−2.94	3.83
50% HFIP (w/w)	5.48 ± 0.02	−2.49	3.80

a Substrate concentration of *ca.* 0.0052 M; binary solvents on a volume-volume basis at 25.0 °C, except for TFE-H_2_O and HFIP-H_2_O solvents which are on a weight-weight basis. T-E are TFE-ethanol mixtures;

b With associated standard deviation;

c Refs [28,29];

d Refs [24,26].

**Table 2.** Correlation of the specific rates of solvolysis of neopentyl chloroformate (this study) and several other chloroformate esters (values from the literature), using the extended Grunwald-Winstein equation (Equation 3).

**Substrate**	***n****^a^*	***l****^b^*	***m****^b^*	***c****^b^*	***l*/*m***	***R****^c^*	***F****^d^*	***Mechanism***
neoPOCOCl	13	1.76 ± 0.14	0.48 ± 0.06	0.14 ± 0.08	3.67	0.977	226	A-E *^e^*
	8	0.36 ± 0.10	0.81 ± 0.14	−2.79 ± 0.33	0.44	0.938	18	I *^f^*
*n-*PrOCOCl *^g^*	22	1.57 ± 0.12	0.56 ± 0.06	0.15 ± 0.08	2.79	0.947	83	A-E
	6	0.40 ± 0.12	0.64 ± 0.13	−2.45 ± 0.27	0.63	0.942	11	I
	8 *^h^*	0.66 ± 0.14	0.91 ± 0.19	−2.61 ± 0.44	0.73	0.912	12	I
EtOCOCl *^g^*	28	1.56 ± 0.09	0.55 ± 0.03	0.19 ± 0.24	2.84	0.967	179	A-E
	7	0.69 ± 0.13	0.82 ± 0.16	−2.40 ± 0.27	0.84	0.946	17	I
MeOCOCl *^g^*	19	1.59 ± 0.09	0.58 ± 0.05	0.16 ± 0.07	2.74	0.977	171	A-E
PhOCOCl *^g^*	49	1.66 ± 0.05	0.56 ± 0.03	0.15 ± 0.07	2.95	0.980	568	A-E
BzOCOCl *^g^*	11	0.25 ± 0.05	0.66 ± 0.06	−2.05± 0.11	0.38	0.976	80	I
*i*-PrOCOCl *^g^*	9	1.35 ± 0.22	0.40 ± 0.05	0.18 ± 0.07	3.38	0.960	35	A-E
	16	0.28 ± 0.04	0.59 ± 0.04	−0.32 ± 0.06	0.47	0.982	176	I
2-AdOCOCl *^g^*	19	0.03 ± 0.07	0.48 ± 0.04	−0.10 ± 0.09	0.06	0.971	130	I
1-AdOCOCl *^g^*	11	0.08 ± 0.20	0.59 ± 0.05	0.06 ± 0.08	0.14	0.985	133	I

a *n* is the number of solvents;

b With associated standard error;

c Multiple Correlation Coefficient;

d *F*-test value;

e Addition-elimnation;

f Ionization;

g See text for references giving the source of this data;

h Calculated for the same eight solvents as are used in the parallel treatment of neopentyl chloroformate solvolyses.
